# Pediatric Brain Tumors: Signatures from the Intact Proteome

**DOI:** 10.3390/ijms23063196

**Published:** 2022-03-16

**Authors:** Diana Valeria Rossetti, Ilaria Inserra, Alessia Nesticò, Federica Vincenzoni, Federica Iavarone, Irene Messana, Massimo Castagnola, Luca Massimi, Gianpiero Tamburrini, Massimo Caldarelli, Claudia Desiderio

**Affiliations:** 1 Dipartimento di Scienze Biotecnologiche di Base, Cliniche Intensivologiche e Perioperatorie, Università Cattolica del Sacro Cuore, 00185 Rome, Italy; dianavaleria.rossetti1@unicatt.it (D.V.R.); ilaria.inserra@gmail.com (I.I.); federica.vincenzoni@unicatt.it (F.V.); federica.iavarone@unicatt.it (F.I.); 2 Fondazione Policlinico Universitario A. Gemelli IRCCS, 00185 Rome, Italy; 3 Istituto di Scienze e Tecnologie Chimiche “Giulio Natta”, Consiglio Nazionale delle Ricerche, 00185 Rome, Italy; imessana53@gmail.com; 4 Department of Chemistry, Sapienza University of Rome, 00185 Rome, Italy; alessia.nestico@libero.it; 5 Laboratorio di Proteomica, Centro Europeo di Ricerca sul Cervello, IRCCS Fondazione Santa Lucia, 00179 Rome, Italy; maxcastagnola@outlook.it; 6 UOC Neurochirurgia Infantile, Dipartimento di Scienze Dell’invecchiamento, Neurologiche, Ortopediche e della Testa-Collo, Fondazione Policlinico Universitario A. Gemelli—IRCCS, Università Cattolica del Sacro Cuore, 00185 Rome, Italy; luca.massimi@policlinicogemelli.it (L.M.); gianpiero.tamburrini@unicatt.it (G.T.); massimo.caldarelli@policlinicogemelli.it (M.C.)

**Keywords:** pediatric brain tumors, proteins, peptides, top-down proteomics, mass spectrometry

## Abstract

The present investigation aimed to explore the intact proteome of tissues of pediatric brain tumors of different WHO grades and localizations, including medulloblastoma, pilocytic astrocytoma, and glioblastoma, in comparison with the available data on ependymoma, to contribute to the understanding of the molecular mechanisms underlying the onset and progression of these pathologies. Tissues have been homogenized in acidic water–acetonitrile solutions containing proteases inhibitors and analyzed by LC–high resolution MS for proteomic characterization and label-free relative quantitation. Tandem MS spectra have been analyzed by either manual inspection or software elaboration, followed by experimental/theoretical MS fragmentation data comparison by bioinformatic tools. Statistically significant differences in protein/peptide levels between the different tumor histotypes have been evaluated by ANOVA test and Tukey’s post-hoc test, considering a *p*-value > 0.05 as significant. Together with intact protein and peptide chains, in the range of molecular mass of 1.3–22.8 kDa, several naturally occurring fragments from major proteins, peptides, and proteoforms have been also identified, some exhibiting proper biological activities. Protein and peptide sequencing allowed for the identification of different post-translational modifications, with acetylations, oxidations, citrullinations, deamidations, and C-terminal truncations being the most frequently characterized. C-terminal truncations, lacking from two to four amino acid residues, particularly characterizing the β-thymosin peptides and ubiquitin, showed a different modulation in the diverse tumors studied. With respect to the other tumors, medulloblastoma, the most frequent malignant brain tumor of the pediatric age, was characterized by higher levels of thymosin β4 and β10 peptides, the latter and its des-IS form particularly marking this histotype. The distribution pattern of the C-terminal truncated forms was also different in glioblastoma, particularly underlying gender differences, according to the definition of male and female glioblastoma as biologically distinct diseases. Glioblastoma was also distinguished for the peculiar identification of the truncated form of the α-hemoglobin chain, lacking the C-terminal arginine, and exhibiting oxygen-binding and vasoconstrictive properties different from the intact form. The proteomic characterization of the undigested proteome, following the top-down approach, was challenging to originally investigate the post-translational events that differently characterize pediatric brain tumors. This study provides a contribution to elucidate the molecular profiles of the solid tumors most frequently affecting the pediatric age, and which are characterized by different grades of aggressiveness and localization.

## 1. Introduction

Brain tumors account for 25% of the malignancies in the pediatric age and are the second-most frequent, following leukemia. Despite the extensive research efforts attempting to elucidate the molecular mechanisms involved in the pathogenesis of brain tumors affecting diverse CNS localizati ons, the processes involved in their development and progression are still far from being understood. 

The present investigation, applying a top-down proteomic approach, addressed the characterization of the intact proteome of tumor tissues of pediatric brain tumors and the profiling of the post-translational molecular events that depict the different histotypes. Targeting its unique information and complementing genetic base studies, proteomics characterizes the molecular phenotype of a cell, tissue, or biological fluid, according to either the gene expression profile or the epigenetic alterations and post-translation modifications (PTMs) that occur in pathological states. Together with its potential for discovering possible biomarkers, clinical proteomics successfully contributes to the comprehension of the molecular mechanisms and events underlying the onset and progression of diseases. 

The first large-scale proteogenomic characterization study of pediatric brain tumors, including medulloblastomas, low- and high-grade astrocytomas, ependymomas, gangliogliomas, craniopharyngiomas, and atypical teratoid rhabdoid histotypes, has been published very recently [1], demonstrating the success and the relevance of “-omic” sciences integration, i.e., genomics, transcriptomics, and proteomics, for the multilayered disclosure of the molecular features of tumors. 

Various review papers, published over the last several years, describe and discuss the advances of proteomic analysis in the field of pediatric brain tumor molecular characterization [2,3,4,5,6]; however, data on the intact proteome have been rarely presented. 

To the best of our knowledge, a comparative proteomic study profiling the undigested proteome of pediatric brain tumor tissues of different tumor grades and locations, the object of the present study, has never been reported. Among the diverse analytical approaches that can be applied for protein and peptide characterization, the investigation of the intact proteome, under the terms of top-down approach, is challenging for studying proteoforms, post-transcriptionally generated, and for identifying the naturally occurring peptidome. The latter includes the “criptides”, i.e., distinguished bioactive peptides generated by in vivo fragmentation of major proteins and exhibiting interesting proper activities [7]. 

The identification of proteoforms associated with specific pathological states could provide the clue to disclose molecular pathways or enzyme activities which, by modifying the structure of proteins/peptides, would influence their functions. These proteoforms could therefore prove to be potential disease biomarkers or molecular targets for tumor therapies.

A previous investigation of tissue pools of medulloblastoma and pilocytic astrocytoma by our group highlighted relevant differences between the profiles of the intact proteome of the two tumors [8] and evidenced potential signatures associated with the differential characterization and distribution of the proteoforms of proteins and peptides of the thymosin family. These findings stimulated the present investigation on individual specimens and an enlarged cohort of samples and tumor histotypes. Therefore, the intact proteome profiles of pediatric brain-tumor tissues of different WHO grades and localization, namely, medulloblastoma (MB), pilocytic astrocytoma (PA), and glioblastoma multiforme (GBM), were compared by label-free LC–MS top-down proteomic analysis to explore qualitative and quantitative variations and to disclose potential signatures and/or biomarkers. The results were also compared with the proteomic data already available on ependymoma [9], resulting in a total of 50 specimens analyzed.

## 2. Results and Discussion

Table 1 lists the proteins and peptides identified in the analyzed tumor tissues, reporting the name, the sequence trait identified (“chain” refers to the entire protein sequence), the theoretical and experimental monoisotopic mass values, and the characterized post-translation modifications (PTMs). As can be observed, the mass values of the elements identified are enclosed in the range of 1.1–22.8 kDa. It is worthy of mention that many of the characterized peptides are protein fragments, possibly produced by in vivo protease activity that can differ from one to another tumor histotype, resulting in different proteomic profiles. Some of these peptide fragments have been characterized in ependymoma pediatric brain tumor, as recently reported [9]. N-terminal acetylation was the main PTM recognized, followed by citrullination, the latter frequently observed in GFAP and vimentin peptide fragments. 

**Figure 1:**
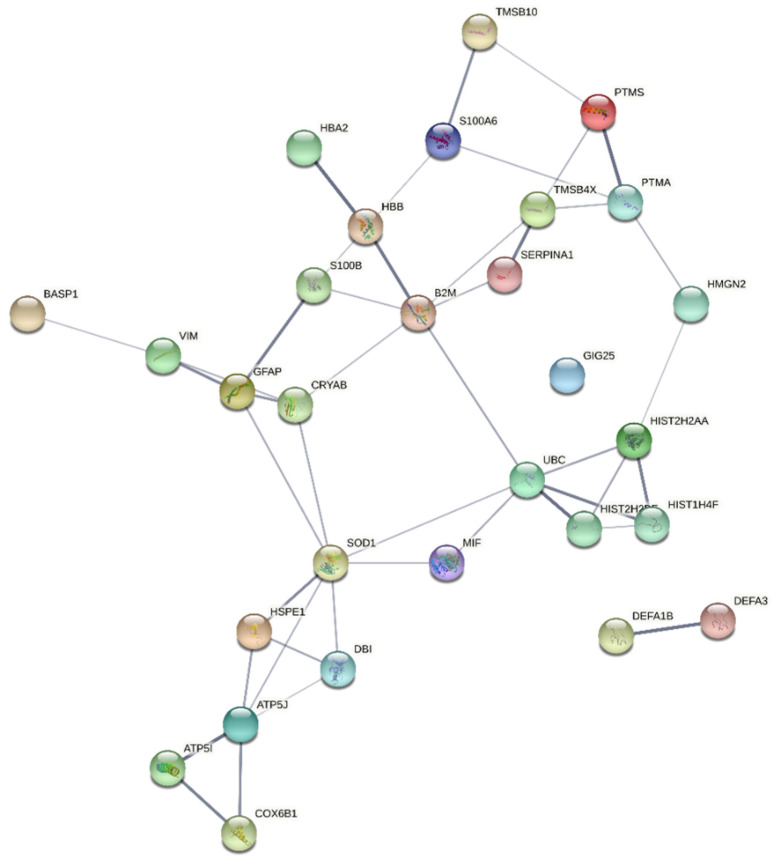
String network (medium confidence 0.400) of both functional and physical associations of the proteins listed in Table 1. Line thickness indicates the strength of data support by confidence.

The protein list in Table 1 was analyzed by a String tool that evidenced a main network of functional interactions with only the α-defensins (DEFA1B, DEFA3) and α-1-antichymotrypsin (GIG25) as disconnected nodes (Figure 1). 

**Figure 2:**
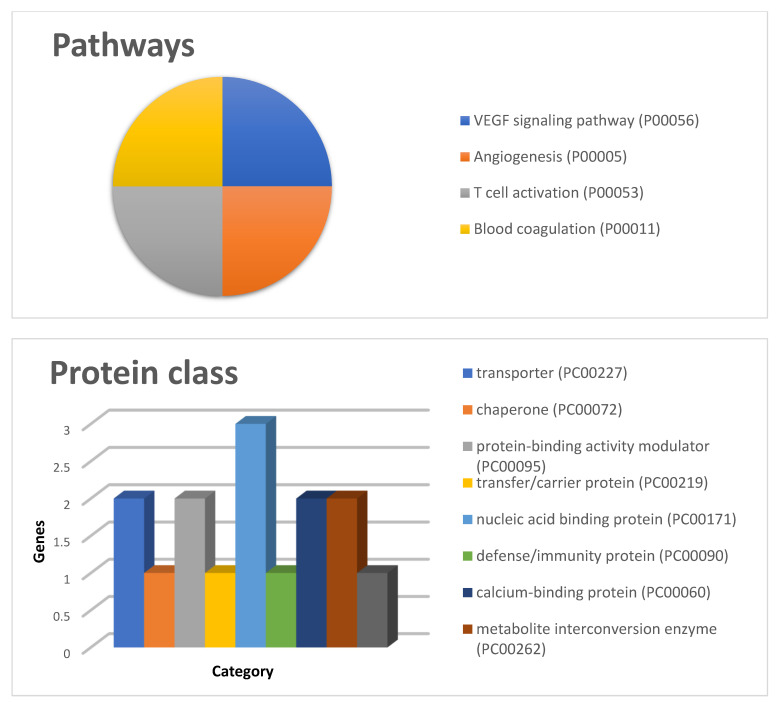
GO pathways and protein class classifications by PANTHER for the proteins listed in Table 1.

Gene ontology analysis using the PANTHER tool classified these proteins into four pathways, namely, VEGF signaling, angiogenesis, T cell activation, and blood coagulation, and into nine protein classes, with the prevalence of the elements belonging to the class of nucleic acid binding proteins (Figure 2). 

Label-free relative quantitation, based on the calculation of the mean peak area value of the XIC plot of three analytical replicates, evidenced significant variations for selected proteins and peptides collectively detected in the tumor specimens that will be discussed in separate paragraphs, based on protein groups. In contrast to the previous investigation [9], ependymoma tumor specimens have been considered in the present study as one group, independently from their cerebral localization. With regard to glioblastoma tumors, separate graphs for male and female patient groups have also been reported since gender differences were observed, in contrast to the other tumors studied.

### 2.1. Proteins and Peptides Belonging to Thymosins’ Family

The elements on which we first focused our attention were the proteins and peptides of the thymosin family, including β- and α-thymosin peptides and parathymosin, due to their potential capability to discriminate medulloblastoma from pilocytic astrocytoma, as resulted from our previous investigation on tumor tissue pools [8]. In the individual specimens analyzed, the thymosin β4 and β10 peptides were commonly detected although they showed different distribution between the tumor histotypes. These peptides are the main sequestering agents of G-actin and are involved in several biological processes, including cell motility and migration, wound healing, and inflammation [14,15]. Both of them have long been studied in relation to tumors showing overexpression and/or association with high-grade malignancies [16]. While thymosin β4 frequently showed overexpression in tumors, the expression of thymosin β10 seemed to be more associated with tumor aggressiveness and metastasis [17,18]. 

**Figure 3:**
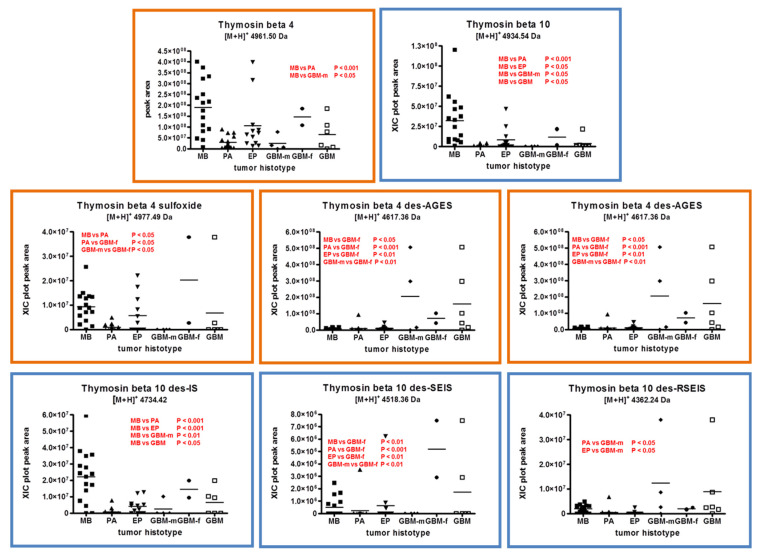
Plot representation of the distribution level of thymosin β4 (orange panels) and thymosin β10 (blue panels) peptides and relative proteoforms, namely, the des-ES and des-AGES C-terminal truncated forms, and the sulfoxide form of thymosin β4; the des-IS, des-SEIS, and des-RSEIS C-terminal truncated forms of thymosin β10 in the analyzed samples, grouped by tumor histotypes (■ medulloblastoma, MB; ▲ pilocytic astrocytoma, PA; ▼ ependymoma, EP; ◆ glioblastoma multiforme–male (GBM-m); ● glioblastoma multiforme-female (GBM-f); □ glioblastoma multiforme, GBM). In each panel, the statistically significant differences between groups with the relative *p*-values, as determined by one-way ANOVA with Tukey’s post-hoc test, reported in red.

Considering first the entire forms of the β-thymosin peptides (Figure 3, upper-line panel), the analysis of individual samples showed higher levels of thymosin β4 ([M+H]^+^ 4961.50 Da, monoisotopic) in MB with respect to PA (*p* < 0.001), confirming the previous findings on tissue pools [8], and with respect to GBM male patients (*p* < 0.05). This result was even more evident for thymosin β10 ([M+H]^+^ 4934.54 Da, monoisotopic), exhibiting higher levels in MB with respect to all other tumor histotypes studied.

The levels of thymosin β4 sulfoxide ([M+H]^+^ 4977.49 Da, monoisotopic), the oxidized form of thymosin β4, distinguished MB from PA and PA from GBM female patients (Figure 3, center-line panels). A difference between female and male GBMs was also recognized, even if the number of subjects and the variance of the data cause the comparison results to be less than robust. 

Together with the entire forms of these peptides, several C-terminal truncated proteoforms have been also characterized. These truncated proteoforms have been identified in our previous studies on the brain tissue of Alzheimer-disease-model mice [10] and pediatric ependymoma [9], craniopharyngioma adamantinomatous [11], medulloblastoma, and pilocytic astrocytoma tumors [8]. 

The des-ES ([M+H]^+^ 4745.43 Da, monoisotopic) and the des-AGES ([M+H]^+^ 4617.36 Da, monoisotopic) thymosin β4, lacking two and four C-terminal amino acid residues, respectively, distinguished female GBMs from the other tumors, the latter showing similar levels instead (Figure 3, center-line panels). 

Regarding thymosin β10, three different C-terminal truncated proteoforms were characterized and quantified, namely, des-IS, des-SEIS, and des-RSEIS thymosin β10 with molecular masses of 4734.42, 4518.36, and 4362.24 Da ([M+H]^+^, monoisotopic), respectively. With respect to all other histotypes, MB showed higher levels of the des-IS form, of statistically significant versus PA and EP (*p* < 0.001). All the other truncated proteoforms showed, in general, comparable levels in all tumors analyzed, with the exception of GBMs, where female and male specimens showed significantly different levels of the des-SEIS and the des-RSEIS proteoforms. The latter showed statistically significant different levels in male GBM with respect to PA and EP (Figure 3, lower panels). 

The results obtained for the C-terminal truncated forms of β-thymosin peptides in single specimen analysis were different from previous findings in tissue pools [8] since the proteoforms in the present study do not distinguish MB from PA, with the exception of thymosin β10 des-IS. 

**Figure 4:**
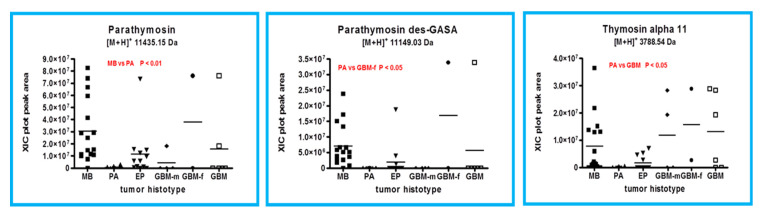
Box plot representation of the distribution level of parathymosin, parathymosin des-GASA, and thymosin α11 in the analyzed samples, grouped by tumor histotype (■ medulloblastoma, MB; ▲ pilocytic astrocytoma, PA; ▼ ependymoma, EP; ◆ glioblastoma multiforme–male (GBM-m); ● glioblastoma multiforme-female (GBM-f); □ glioblastoma multiforme, GBM). In each panel, the statistically significant differences between groups with relative *p*-values, as determined by one-way ANOVA with Tukey’s post-hoc test, are reported.

Parathymosin protein ([M+H]^+^ 11,435.15 Da, monoisotopic) and its des-GASA C-terminal truncated form ([M+H]^+^ 11,149.03 Da, monoisotopic), both determined in the tumor tissues analyzed, also belong to the thymosin family. The relative quantitative analysis of parathymosin and its des-GASA truncated form and of thymosin α11 in all tumor tissues analyzed is illustrated in Figure 4. The entire form of parathymosin resulted as overexpressed in MB with respect to PA (*p* < 0.01), and the des-GASA proteoform distinguished PA from female GBMs. Thymosin α11, as well as thymosin α1, is a bioactive peptide fragment of prothymosin α [7], a protein not detected in the analyzed samples in its entire form. Thymosin α11 ([M+H]^+^ 3788.54 Da, monoisotopic) distinguished PA from GBM (*p* < 0.05), while thymosin α1 did not show significantly different levels within all of the tumor histotypes. 

### 2.2. Ubiquitin and Truncated Proteoforms

**Figure 5:**
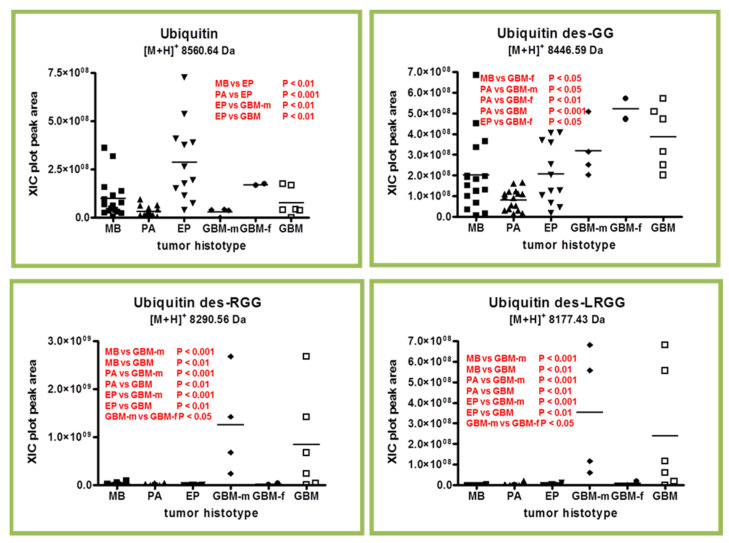
Box plot representation of the distribution levels of ubiquitin and its C-terminal truncated forms in the analyzed samples grouped by tumor histotype (■ medulloblastoma, MB; ▲ pilocytic astrocytoma, PA; ▼ ependymoma, EP; ◆ glioblastoma multiforme–male (GBM-m); ● glioblastoma multiforme-female (GBM-f); □ glioblastoma multiforme, GBM). In each panel, the statistically significant differences between groups with relative *p*-values as determined by one-way ANOVA with Tukey’s post-hoc test are reported.

In analogy to thymosins, for ubiquitin protein several C-terminal truncated proteoforms were determined in the analyzed tumor tissue in addition to the entire chain. These truncated proteoforms have been characterized, together with truncated β-thymosin peptides, in our previous studies [8,9,10,11]. These proteoforms lack two, three, or four C-terminal amino acid residues generating the des-GG, des-RGG, and des-LRGG truncated forms, with the des-GG generally resulting in the most abundant and frequently observed form. As shown in the box plots of Figure 5, both the ubiquitin and its truncated proteoforms showed statistically significant differences in distribution levels within the tumor histotypes, with high levels of ubiquitin particularly distinguishing EP from the other histotypes. Particularly significant was the difference between EP and PA (*p* < 0.001). 

A significant difference was also found between EP, GBM and male GBM. Interestingly, the truncated forms of ubiquitin showed a significant difference in GBM versus all other tumors, the des-GG results being particularly higher in female GBMs, while, conversely, the des-RGG and the des-LRGG results were higher in male GBMs. 

As with C-terminal truncated thymosins, the origin and biological role of the ubiquitin C-terminal truncated forms in the brain tumor tissue proteome is still unclear; however, all together, they could be the phenotypic expression of the action of proteolytic enzymes that varies between the diverse histotypes. 

### 2.3. β-Thymosins and Ubiquitin Proteoforms Distribution in Tumor Histotypes

**Figure 6:**
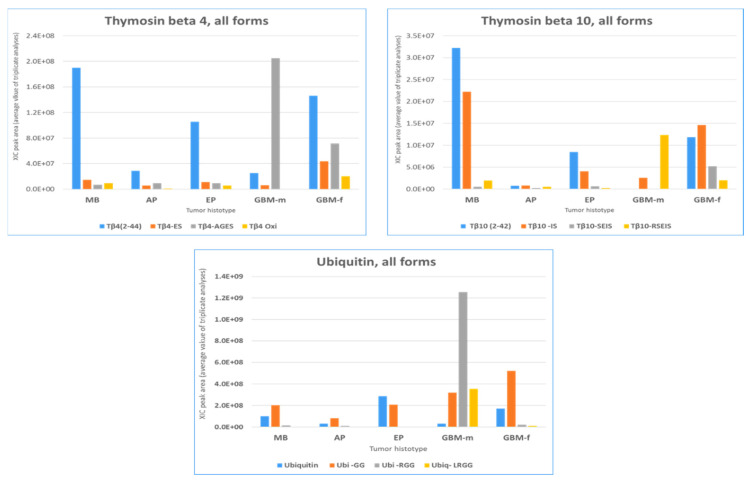
Relative distributions of thymosin β4, thymosin β10, ubiquitin, and their C-terminal truncated proteoforms as resulting from XIC plot peak-area relative quantitation and considering the average values of the three analytical replicates for each element.

In addition to evaluate the relative quantitation of thymosins and ubiquitin proteoforms in tumor tissues, it was also interesting to depict their relative distribution by tumor histotype (Figure 6). Regarding thymosin β4, the entire peptide (Tβ4 2-44) was the prevalent form in MB, PA, EP, and GBM-f, with particular evidence in MB. The des-AGES form was instead predominant in GBM-m. In contrast to MB, PA, and EP, GBM, and especially GBM-f, characteristically showed a consistent presence of thymosin β4 and β10 truncated forms. Their different distributions in male and female GBMs again establish their different protein profiles. MB and EP showed a similar distribution pattern for thymosin β10 proteoforms. It is interesting to underline the detection of the des-SEIS and des-RSEIS forms of thymosin β10 in GBM, which are, on the contrary, rarely observed, or observed at very low levels, in the other tumors.

The des-GG form of ubiquitin was quantitatively characterized in MB, PA, and EP tumors at levels more or less comparable to the entire form. Interestingly, in GBM, the levels of all ubiquitin truncated forms far exceeded those of the entire form. Of further notice, male GBMs were characterized by the des-RGG and des-LRGG forms, generally unfrequently observed or detected at very low levels, that were also prevalent over the des-GG form. Additionally, for ubiquitin, the pattern of the distribution of the truncated proteoforms was different in male and female GBMs.

The peculiar distribution of thymosins and ubiquitin proteoforms in pediatric brain tumors was intriguing and needs further investigation aimed at disclosing their origins and different biological functions, following their molecular structure modifications. 

### 2.4. Other Protein Elements

**Figure 7:**
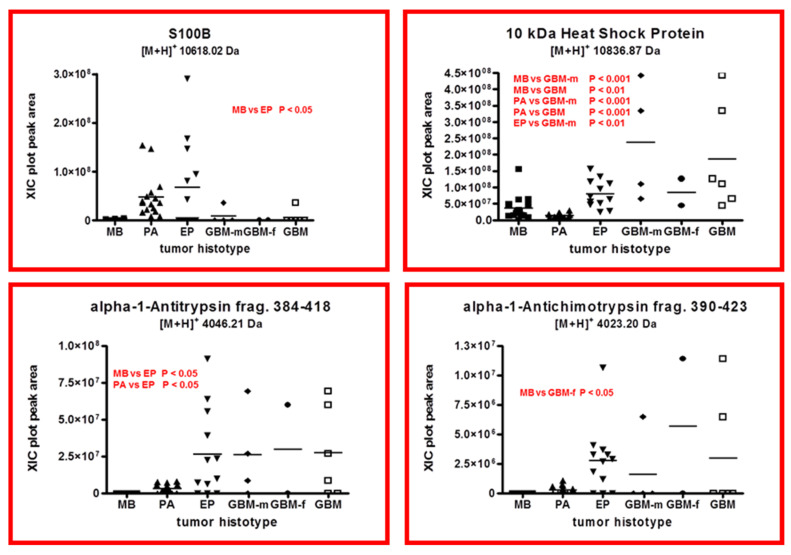
Box plot representation of the distribution level of S100B, 10 kDa heat shock protein, 384-418 fragment of α-1-antitrypsin, and 390-423 fragment of α-1-antichimotrypsin, in the analyzed samples, grouped by tumor histotype (■ medulloblastoma, MB; ▲ pilocytic astrocytoma, PA; ▼ ependymoma, EP; ◆ glioblastoma multiforme–male (GBM-m); ● glioblastoma multiforme-female (GBM-f); □ glioblastoma multiforme, GBM). In each panel, the statistically significant differences between groups with relative *p*-value as determined by one-way ANOVA with Tukey’s post-hoc test are reported.

Figure 7 reports the box plot of S100B ([M+H]^+^ 10,618.02 Da, monoisotopic), 10 kDa heat shock protein ([M+H]^+^ 10,836.87 Da, monoisotopic), and fragments 384-418 ([M+H]^+^ 4046.21 Da, monoisotopic) and 390-423 ([M+H]^+^ 4023.20 Da, monoisotopic) of the α-1-antitrypsin and α-1-antichimotrypsin, respectively.

PA and EP showed higher levels of S100B with respect to the other tumors, and the difference between EP and MB was statistically significant (*p* < 0.05). S100B protein was investigated in newly diagnosed gliomas, and no correlation between protein levels and patient prognosis was observed [12]. In accordance with this observation, in the present study, the gliomas of lower WHO tumor grade, i.e., PA and EP, showed higher levels of S100B.

The 10 kDa heat shock protein showed markedly higher levels in male GBM, and that influenced the total GBM plot. The lowest levels of this protein were found in PA, the tumor of lower WHO grade. This could be in accordance with the reported overexpression of this protein in cancer and with its multifaceted role, which includes the inhibition of apoptosis [12,13]. 

Both the fragments of α-1-antitrypsin showed very low levels in MB, while similar quantities were recognized in EP and GBM. Particularly, the fragment 384-418 of α-1-antitrypsin distinguishes EP from MB and PA. In contrast, the fragment 390-423, although showing apparent different levels in MB, PA, and EP, did not exhibit differences of statistical significance, but it does distinguish MB and GBM-f (*p* < 0.05). 

**Figure 8:**
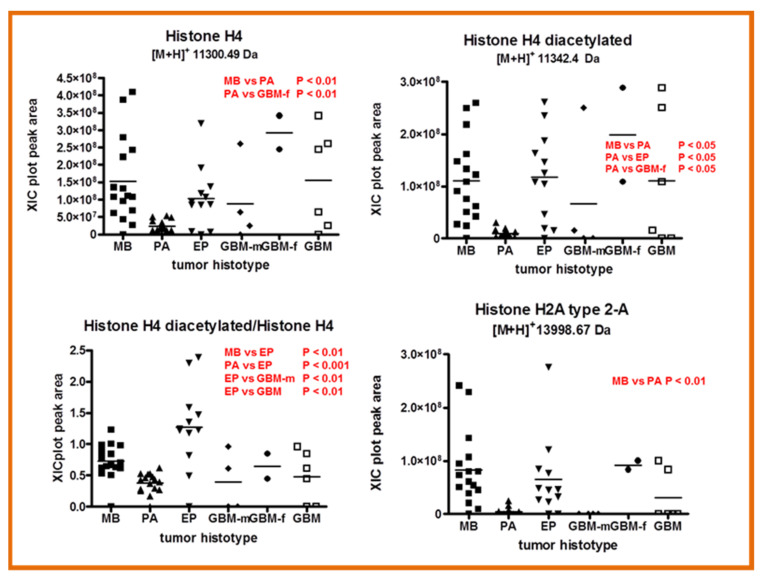

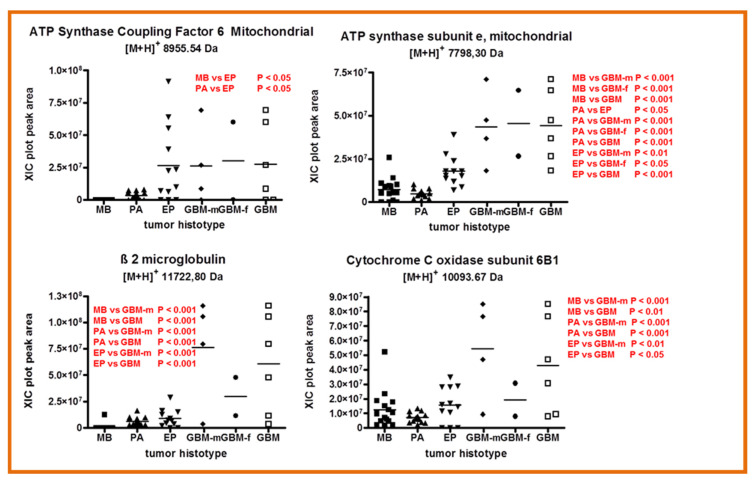
Box plot representation of the distribution level of Histone H4, Histone H4 diacetylated form and their ratio, Histone H2A type 2-A (**upper panel**), and ATP synthase coupling factor 6, ATP synthase subunit e, and cytochrome C oxidase subunit 6B1 mitochondrial proteins and β2 microglobulin (**lower panel**) in the analyzed samples grouped by tumor histotype (■ medulloblastoma, MB; ▲ pilocytic astrocytoma, PA; ▼ ependymoma, EP; ◆ glioblastoma multiforme–male (GBM-m); ● glioblastoma multiforme-female (GBM-f); □ glioblastoma multiforme, GBM). In each panel, the statistically significant differences between groups with relative *p*-values, as determined by one-way ANOVA with Tukey’s post-hoc test are reported.

Histone H4, its diacetylated form, and histone H2A type 2-A showed different levels in MB and PA. Histone H4 also distinguished PA from GBM-f, the latter showing higher levels of the protein (Figure 8, upper panels). The levels of H4’s di-acetylated form were significantly lower in PA (WHO grade I) with respect to MB (WHO grade IV) and EP (WHO grades II and III) posterior fossa tumors. However, considering the ratio between the diacetylated and the unmodified forms of histone H4, the results were different, i.e., MB and PA levels were not any more statistically different, while EP showed significantly higher levels with respect to all of the other tumors. 

In summary, histone H4 diacetylation strongly characterized EP tumors. Likewise, the levels of histone H4 and histone H2A type 2-A were lower in PA with respect to all other histotypes and either distinguished MB or PA. The modifications of histones at their tails regulate gene expression and therefore different cellular processes. The acetylation/deacetylation processes of histones are involved in the regulation of DNA transcription and were found misbalanced in different cancer diseases and gliomas [19,20], and for that reason, they are now being explored for new pharmacological treatments [21].

The lower panel of Figure 8 reports the box plots and the statistical results of mitochondrial proteins and β2 microglobulin. Mitochondrial ATP synthase coupling factor 6 distinguished MB and PA from EP, the latter exhibiting higher levels in comparison. ATP synthase subunit e generally showed similar levels in MB, PA, and EP posterior fossa tumor, but higher levels in GBM. β2 microglobulin and cytochrome C oxidase subunit 6B1 showed a similar trend and significantly higher levels of m-GBM, again confirming differences between male and female GBM profiles. These results are in accordance with the reported overexpression of cytochrome C oxidase subunit 6B1 in gliomas, which accomplishes mitochondrial metabolic remodeling in this disease, and it is involved in apoptosis inhibition, mitochondrial function modulation, and stress resistance [22]. 

### 2.5. Vimentin and Glial Fibrillary Acidic Protein Fragments: PTMs Characterization 

**Figure 9:**
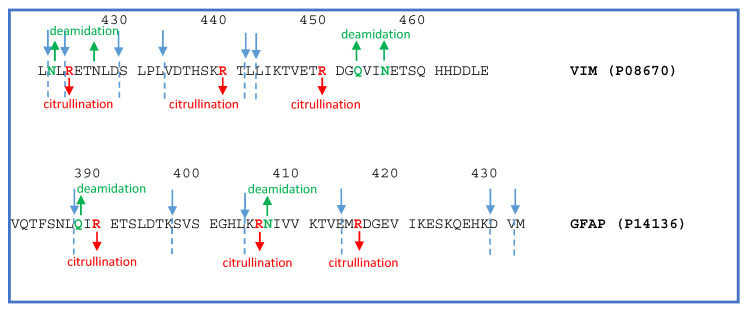
Vimentin (VIM) and glial fibrillary acidic protein (GFAP) C-terminal sequences showing the cleavage sites (blue color) generating the identified peptide fragments and the position of citrullination and deamidation PTMs.

A separate paragraph is dedicated to the naturally occurring peptide fragments of vimentin (VIM) and glial fibrillary acidic protein (GFAP) identified in the tumor tissue analyzed, often carrying citrullination and/or deamidation PTMs (corresponding to delta mass shifts of +0.9840276 and +0.9840 Da, respectively) (Table 1). Figure 9 illustrates the C-terminal sequences of VIM and GFAP proteins with the annotation of the cleavage sites (blue color), generating the identified peptide fragments, and of the position of citrullination (red color) and deamidation (green color) PTMs, the latter identified by both manual inspection and theoretical and experimental comparison of the tandem MS spectra. As shown in Table 1, in some cases it was not possible to assign the position of the PTM. Therefore, for some peptides, alternative PTM positions were indicated for the mono- and poly-citrullinated peptides. These fragments, with the exception of the peptides 41-59, 15-38, and 398-430 for GFAP and 54-69 of VIM, have been characterized in EP tumors in our previous investigation [9], which underlined the presence of the PTM characteristically at the Arg residue inside the sequence trait—KTVETrDG—in vimentin and—KTVEMrDG—in GFAP.

It can be observed in Figure 9 that the peptide fragments are generated, especially in VIM, by the cleavage at N-terminal Leu residues. For GFAP, different sites of cleavage were instead observed. 

**Figure 10:**
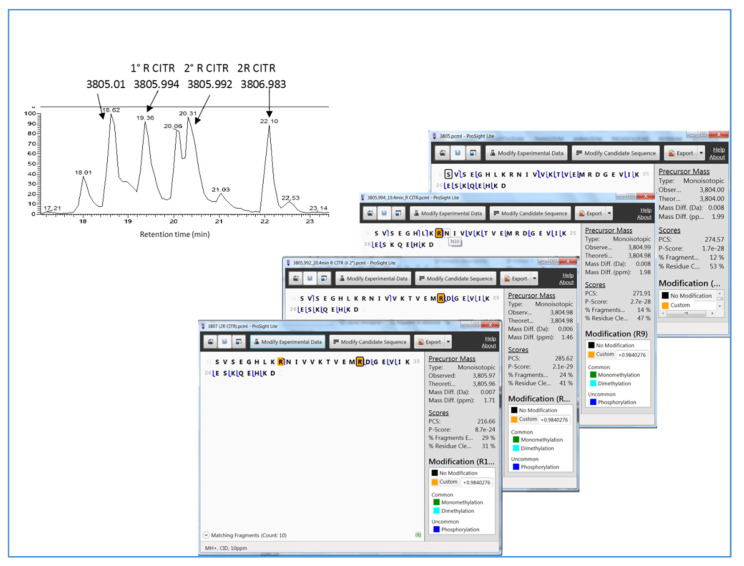
Enlarged view of the LC–MS chromatographic separation of the GFAP peptide fragment 398-430 ([M+H]^+^ 3805.01 Da) and its citrullinated forms. The peptide fragments’ identification by Prosight Light comparison of the experimental and theoretical tandem MS data is also reported.

In accordance with arginine deamidation modification, citrullinated peptides exhibited longer chromatographic retention times with respect to the unmodified form, with higher numbers of citrullination PTMs indicating stronger retention on the stationary phase. It is further noteworthy that mono-citrullinated peptides showed different elution times depending on the position of the PTM, evidencing a strong influence of the position of the modified Arg residue on the physico-chemical properties of the peptide. An example is reported in Figure 10, where the enlarged view of the LC–MS chromatographic profile of the peptide fragment 398-430 of GFAP ([M+H]^+^ 3805.01, Da, monoisotopic) and its citrullinated forms is shown. The different position of the PTM in the mono-citrullinated peptides ([M+H]^+^ 3805.99 Da, monoisotopic) at R_406_ or R_416_ residues produced a retention time difference of about 1 min, the peptide with modification at R_406_ eluting first. The longest retention time was observed for the di-citrullinated form, showing a retention time shift with respect to the unmodified peptide of about 3.5 min. In the figure, the identification data by comparison of the theoretical and experimental tandem MS spectra are also reported. 

It is interesting to comment on the peculiar distribution of the C-terminal fragments of vimentin and GFAP in the different tumor tissues analyzed (Supplementary Figure S1A,B). In particular, it is worthy of mention that most of the VIM and GFAP fragments were detected with high frequency in PA and EP tumors, while they were not detected or were detected at negligible levels in MB tumors.

Considering the WHO grade of the studied tumors, it seems that VIM and GFAP fragmentation is associated with malignancies of lower severity. For GBM tumors, WHO grade IV, a separate consideration is due. GFAP fragments were undetected in GBMs, with the exception of very low levels of the peptide 398-430 in f-GBMs, thus confirming the occurrence of a lower degree of fragmentation of GFAP in high-grade tumors. In contrast, VIM peptide fragments were observed in GBM, and differences between m- and f-GBMs were recognized. The peptides were generally detected in f-GBMs and undetected in m-GBMs, again evidencing a potential sex dimorphism of pediatric GBM disease. This observation seems to be in agreement with the better overall survival after treatment and the lower incidence of GBM disease in females compared to males [23,24,25], thus supporting the suggestion that the greatest protein fragmentation is observed in less aggressive forms of brain tumors.

One topic of discussion is the time schedule of protein citrullination and protein fragments generation in vivo. It would be very interesting to establish the sequence of these processes and their mutual influences. However, protein citrullination PTM has been studied in relation to several pathologies, including autoimmune diseases, inflammation, tumor onset and progression [26,27,28,29], and neurodegenerative diseases [30,31]. In relation to brain tissue, vimentin and GFAP citrullination is produced by the protein-arginine deiminase type-2 (PAD2) enzyme [30,32]. PAD enzymes’ overexpression and citrullination PTM were therefore the object of several research studies on cancer [33,34]. Wang et al. have reviewed the roles of PAD2- and PAD4-mediated protein citrullination in various forms of cancers. These enzymes can have an opposite role, either promoting tumor development or reducing its malignancy, depending on tumor localization and on the pathway affected [35]. Activated Jurkat cells overexpressing the PAD2 enzyme showed apoptotic features together with an increased citrullination of proteins, including VIM. The PAD2-induced apoptosis process seemed, therefore, to involve VIM, with a role in the cell-surface and extracellular environments in the mechanism of autoantigen presentation to the immune system and in the apoptotic mechanisms of activated T lymphocytes [32]. On the other hand, a correlation between PAD2 activation/VIM citrullination and neuroinflammation was shown, citrullinated VIM resulting as an indicator of astrocytes’ reactive state [36].

The catalytic activity of PAD enzymes, and therefore protein citrullination PTM, is dependent on calcium concentration. Particularly, it requires high intracellular calcium concentrations, which are only achievable following cell membrane disruption or the apoptosis and autophagy processes [37,38,39]. Although VIM is an intracellular protein, under distinctive conditions, the protein was found on the cell surface [40,41]. It is therefore still unclear what the role and the occurrence of VIM are, as well as that of its citrullinated form in the intra- and extra-cellular environment and its cleavage processes. We also find interesting the role of citrullination PTM in the stimulation of the immune response through the mechanism of antigen processing and presentation by citrullinated peptides/MHC complex [42,43]. The immunological consequences of citrullination PTM are therefore the object of numerous studies considering how PAD enzymes act in different cell types, including neutrophils, monocytes, and macrophages. Brentville et al. demonstrated that citrullinated VIM epitopes on tumor cells are the targets of CD4 T cells, resulting in strong antitumor responses [44]. Therefore, an immunogenic citrullinated peptide vaccine in transgenic mouse models of melanoma and ovarian cancer was developed [45]. 

As described above, C-terminal VIM peptides were identified in the present study in EP and PA tumor tissues, many of which show citrullination PTMs and enclose the sequence trait 447–455—VETRDGQVI—. It is interesting that, in immunotherapy clinical trials, autologous modified dendritic cells were exposed to citrullinated peptides, including VIM peptides presenting the same sequence we identified, and immunoregulatory and anti-inflammatory effects in relation to rheumatoid arthritis were observed [46]. 

### 2.6. Hemoglobin

Based on our previous investigations [9,47] and on the intriguing relationships between hemoglobin and brain tumors [48], special attention was paid to the relative quantitation of hemoglobin chains in the tumor tissues analyzed. 

As reported in Table 1, in addition to the identification and quantitation of α- and β-hemoglobin chains, an interesting finding was the characterization of the hemoglobin α-chain missing the C-terminal arginine ([M+H]^+^ 14,961.79 Da, monoisotopic) (des-Arg αHb). Des-Arg αHb was characterized in GBM samples and sequenced following tandem mass spectrometry experiments using both CID and HCD techniques of fragmentation (Supplementary Figure S2). 

**Figure 11:**
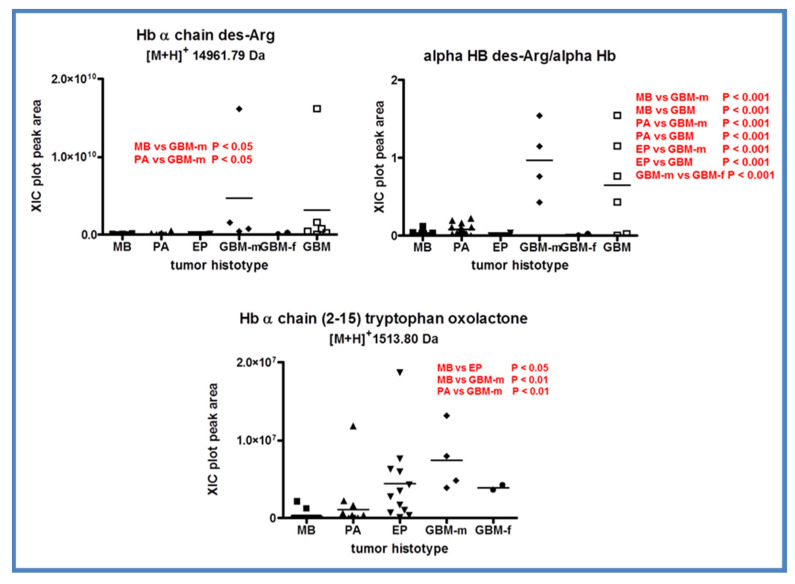
Box plot representation of the distribution level of des-Arg αHb (**upper left panel**), of the ratio des-Arg αHb/αHb (**upper right panel**), and of αHb fragment 2-15 with C-terminal tryptophan oxolactone PTM (**lower panel**) in the analyzed samples grouped by tumor histotype (■ medulloblastoma, MB; ▲ pilocytic astrocytoma, PA; ▼ ependymoma, EP; ◆ glioblastoma multiforme–male (GBM-m); ● glioblastoma multiforme-female (GBM-f); □ glioblastoma multiforme, GBM). In each panel, the statistically significant differences between groups with relative *p*-values as determined by one-way ANOVA with Tukey’s post-hoc test are reported.

The relative quantitation of des-Arg αHb disclosed interesting differences among the tumor histotypes investigated (Figure 11). Des-Arg αHb showed higher levels in m-GBMs over MB and PA (*p* < 0.05), also influencing the level of the total GBMs plot. This effect was even more evident when the ratio of des-Arg αHb/αHb peak areas was depicted. This ratio was significantly higher in m-GBMs with respect to all other tumors analyzed and to f-GBMs (*p* < 0.001), again confirming sex differences in the pediatric GBM protein profile. In contrast, α- and β-Hb chains did not show significantly different levels between the tumor histotypes (data not shown). The formation of des-Arg αHb therefore remains an event to be clarified and needs deeper investigation at the genomic and proteomic levels.

The identification of this truncated form of αHb chain in brain tumor tissues, selectively marking m-GBMs over the other tumor histotypes, is intriguing due to its peculiar properties. Des-Arg αHb was identified a long time ago in the plasma and urine of patients with acute hemolysis of different origins [49] and in favism [50] as a product of the action of a plasma carboxypeptidase. The enzyme was reported to act on the free αHb generated by the massive hemolysis, and the des-Arg αHb truncated form produced was called Koelliker hemoglobin. The des-Arg αHb was later identified in hemoglobin extract from human placenta for potential use as a blood substitute. It was supposed that it was generated by the action of a carboxypeptidase during the preparation of frozen placenta tissue extract. Interestingly, this hemoglobin extract exhibited higher oxygen affinity and no effects from classical hemoglobin effectors, as a consequence of the α-chain C-terminal cleavage. In fact, the addition of an inhibitor of the enzymatic cleavage produced a hemoglobin extract with normal oxygen binding properties [51]. Later, des-Arg αHb was demonstrated to enhance the dissociation of the hemoglobin tetramer to dimer, to show higher oxygen affinity by simultaneously diminishing the cooperativity of the binding, and to show in rats more vasoconstrictive properties than the entire chain [52]. Therefore, the C-terminal Arg_141_ in the αHb chain demonstrated having an important role in maintaining either the tetrameric structure of hemoglobin or in its normal oxygen affinity and vasoconstrictor properties. In this paper, the production of des-Arg αHb was possibly ascribed to the action of carboxypeptidase N or M [52], the latter described as abundant in placental brush border membrane and able to remove the C-terminal arginine residue more efficiently. 

To the best of our knowledge, this is the first time the des-Arg αHb has been identified as naturally occurring in tissue homogenates, and the differences observed in male and female GBMs are interesting and require future investigation. Male and female GBMs are reported as biologically distinct diseases, outlining the importance of forthcoming studies in this respect in view of a personalized medicine approach [23,24,25,53].

The fragment 2-15 of the Hb α-chain carried oxidation PTM at the C-terminal Trp (oxolactone) and showed a different distribution within the tumor histotypes with significantly higher levels in EP and m-GBMs (Figure 11, lower panel). Trp residues represent the target sites of oxidation PTM, and therefore play an important role inside proteins, especially in tissue exposed to oxygen reactive species, such as skeletal muscle or mitochondria [54,55]. A modification at Trp_15_ of the hemoglobin β-chain with a delta mass of +14 Da was identified after the treatment of the protein with hydrogen peroxide, evidencing this amino acid site as a target of protein oxidation [54]. Later, Trp oxidation was also identified in actin and troponin 1 in rat skeletal muscle under oxidative stress conditions [55]. PTMs, such as selective oxidation of amino acid residues along the sequence, modify protein structure and function and can also be prodromal to protein chain fragmentation [56,57]. On this basis, with regard to the peptide αHb 2-15, the question arises as to whether it is the oxidation of Trp_15_ that causes the protein cleavage, generating the peptide fragment, or it is the protein cleavage that makes the amino acid residue more sensitive to oxidation.

## 3. Materials and Methods

### 3.1. Chemicals

The 2,2,2-trifluoroacetic acid (TFA) and the sodium chloride were from Mallinckrodt Baker B.V. (Deventer, The Netherlands) and Fluka (Sigma–Aldrich Chemie GmbH, Buchs, Switzerland), respectively. Acetonitrile (ACN), methanol (MeOH), and formic acid (FA), all of LC–MS grade, were purchased from Merck (Darmstadt, Germany). Dye reagent (Comassie Brilliant Blue G-2509) was purchased from Bio-Rad Laboratories (Hercules, CA, USA). Bovine serum albumin (BSA) and protease inhibitor cocktail (AEBSF, aprotinin, bestatin, E-64, EDTA, and leupeptin) were from Sigma–Aldrich (St. Louis, MO, USA). 

### 3.2. Instrumentation

Tissue homogenization and sonication were carried out by means of a Wheaton^®^ 903475 Overhead Stirrer apparatus (Wheaton, Millville, NJ, USA) and a Branson Sonifier 450 (Branson Ultrasonics, Danbury, CT, USA), respectively. Total protein concentration was determined in duplicate by Bradford assay (Bio-Rad Laboratories, Hercules, CA, USA) and UV–Vis spectrophotometer (8453 UV–Vis Supplies, Agilent Technologies, Waldbronn, Germany) detector using BSA as the protein of reference. For sample centrifugation, a thermostated centrifuge SL16 R (Thermo Fisher Scientific, Langenselbold, Germany) or Mini Spin (Eppendorf AG, Hamburg, Germany) were used as specified for sample treatment. HPLC–ESI–MS/MS analyses were performed on an UltiMate 3000 RSLCnano System (Dionex, Sunnyvale, CA, USA) coupled with an Orbitrap Elite MS detector with ESI or EASY-Spray nanoESI sources (Thermo Fisher Scientific), as specified elsewhere.

### 3.3. Sample Collection and Treatment

Tumor tissues were obtained from 50 pediatric patients affected by medulloblastoma (*n* = 16), pilocytic astrocytoma (*n* = 16), ependymoma (*n* = 12), and glioblastoma (*n* = 6), who underwent the surgical removal of the tumor at the Pediatric Neurosurgery Complex Operational Unit of Fondazione Policlinico Universitario Agostino Gemelli IRCCS. Tumor tissues were collected during surgery under sterile conditions and immediately stored at −80 °C. The study was realized under the approval of the local Ethical Committee (Prot.N 0034878/16 ethics code). Table 2 lists the specifications of the tumor tissues analyzed, including grade classification, tumor localization, WHO grade, and diagnosis data.

Tissue samples were thawed on ice, washed with cold phosphate-buffered saline solution (PBS) containing the protease and phosphatase inhibitor cocktail, and weighed. Tissues were added of a volume of water/ACN solution (70/30, *v*/*v*), containing 0.4% TFA (*v*/*v*) and protease inhibitor (1:100, *v*/*v*), in order to have a final concentration tissue/solution of 0.2 mg/µL per sample, homogenized and sonicated for 3 × 1 min cycles. Following centrifugation at 24,000× *g* for 30 min at 4 °C, the resulting acid-soluble fraction was collected for LC-MS proteomic analysis.

### 3.4. LC-MS Proteomic Analysis

LC–MS proteomic analysis was performed in triplicate at a thermostated temperature of 40 °C on a Zorbax 300 SB-C8 (3.5 µm, 1.0 i.d., ×150 mm) (Agilent Technologies, (Santa Clara, CA, USA) chromatographic column coupled with an Acclaim PepMap300 trap cartridge (Thermo Fisher Scientific) as already described in our previous paper [9]. Briefly, elution was performed in step gradient mode using eluent A (FA 0.1%, *v*/*v*) and eluent B (water/ACN 20:80, *v*/*v*, 0.1% FA, *v*/*v*) as following: (step 1) from 0% to 2% B (2 min), (step 2) from 5% to 70% B (38 min), (step 3) from 70% to 99% B (5 min), (step 4) from 99% to 5% B (2 min), (step 5) 5% B (5 min) at a flow rate of 50 μL/min. The samples were diluted with 0.1% (*v*/*v*) FA aqueous solution to allow the injection of 7.8 μg of total proteins in 20 μL of injection volume. The Orbitrap Elite MS instrument operated in positive ionization mode at a resolution of 60,000 in 350–2000 *m*/*z* scan filter range in data-dependent scan (DDS) mode, performing MS/MS fragmentation of the 5 most-intense signals of each full-scan MS spectrum by high-energy collisional dissociation (HCD) mode. The minimum signal was set at 500.0 and the isolation width at 5.00 *m*/*z*. Normalized collision energy was set at 35.0. Capillary temperature was 300 °C, and the source voltage was +4 kV. Acquisition started at 4 min in order to avoid salt-source contamination in the first minutes of elution.

### 3.5. Data Analysis

LC–MS proteomic data were elaborated by the Xcalibur software (version 2.0.7 SP1, Thermo Fisher Scientific) by both manual inspection and Proteome Discoverer 1.4 software (version 1.4.1.14, Thermo Fisher Scientific) elaboration. ExPASy UniProtKb database and proteomics tools (http://www.expasy.org/tools/) (accessed on 10 January 2022) were used for protein characterization and ProSight Lite v1.4 free software [58] for experimental/theoretical spectra matches, tandem MS spectra and PTM annotations. A String tool was used to investigate both functional and physical protein association networks [59]. Gene Ontology (GO) classification was performed using the Protein Analysis THrough Evolutionary Relationships (PANTHER http://www.pantherdb.org) (accessed on 10 January 2022) classification system (version 16.0) [60], using Fisher’s exact test and the correction of the false discovery rate (FDR). Label-free relative quantitation of the proteins/peptides was assessed by comparing the peak area values (signal/noise ratio >5) of the extracted ion current (XIC) plots, obtained by extraction of the ion current signals of the relative multiple charged ions (*m*/*z*) from the total ion current (TIC) profile. Significant differences in protein quantitative levels between samples were calculated by one-way ANOVA with Tukey’s post-hoc test, considering *p*-values < 0.05 as significant.

## 4. Conclusions

To the best of our knowledge, the present investigation illustrates the first comparative proteomic study of pediatric brain tumor tissues of different WHO grades and brain region locations following a LC–MS top-down approach driven by the characterization of the intact proteome. Together with full-sequence proteins and peptides, several peptide fragments have been identified, often carrying modifications such as acetylation, oxidation, citrullination, and deamidation PTMs, N- and C-terminal cleavages, and truncations. Label-free relative quantitation evidenced different levels of selected proteins and peptides and/or their proteoforms in the tumor tissues analyzed, evidencing different proteomic profiles associated with the diverse brain tumor histotypes studied. Top-down proteomics is the tool of excellence for PTM identification and for studying the peptidome and the naturally occurring protein fragmentome. The addition of a proteases inhibitor cocktail, high performance mass spectrometry, and analytical replicates ensured that we obtained reliable and reproducible results. Protein identification was accomplished by both the manual inspection of the MS/MS spectra and the comparison of the experimental and theoretical datasets, taking into account the difficulty in validating the presence of proteoforms, truncated forms, protein fragments, and cryptides by immunochemical methods, as they may lack such specificity.

Distinct patterns of protein and peptide proteoforms, and particularly of C-terminal truncated forms of beta thymosins and ubiquitin, marked the tumor histotypes differently. It is noteworthy to observe that proteins and peptides of the thymosin family generally showed very low levels in PA with respect to all other histotypes in accordance with previous findings [8], whereas increased levels were confirmed to be associated with tumors of the higher WHO grades. PA was characterized by the identification of numerous C-terminal peptide fragments of VIM and GFAP, frequently carrying citrullination and deamidation PTMs. These fragments have been also identified in EP—however with a prevalent distribution in the WHO grade II specimens. A separate study will be devoted to deeply investigating the occurrence and distribution of proteins’ citrullination PTM inside pediatric brain tumors, with particular regard to VIM and GFAP peptide fragments and to glial tumors. Inside PA, EP, and GBM glial tumors, differences have been observed according to tumor grade. While S100B was frequently detected in EP and PA, the protein was instead undetected or detected at low levels in MB and GBM, therefore resulting in, or possibly more associated with, tumors of lower grades of aggressiveness.

MB, the most frequent aggressive brain tumor of the pediatric age, WHO grade IV, is, unusually, of embryonic origin. MB showed higher levels of thymosin β4 and β10 peptides over the other tumors studied, with thymosin β10 in both its entire and des-IS forms particularly marking this tumor histotype.

Tumor tissues of GBM, a glial tumor of WHO grade IV, were peculiar for the characterization of a truncated form of αHb chain lacking the C-terminal Arginine (Arg_141_) that particularly marks m-GBMs. This truncated form exhibits oxygen affinity and vasoconstrictive properties different from those of the intact chain, raising the need to deeply investigate its role and function in GBM in future studies. Male GBM specimens were also characterized by the identification of the shorter C-terminal truncated proteoforms of thymosin β10 and ubiquitin, i.e., des-RSEIS thymosin β10 and des-LRGG ubiquitin, unfrequently observed in the other tumors studied. An interesting finding, however, taking into account the very few samples analyzed, was the dissimilar proteomic profile of m- and f-GBM tissues, in agreement with the literature data outlining sex dimorphisms in pediatric GBM disease.

The study of the undigested proteome allowed us to draw conclusions, providing the first overview of the different proteoforms that characterize pediatric brain tumor histotypes of different locations and grades of aggressiveness and to depict peculiar molecular profiles of the solid tumors most frequently affecting the pediatric age.

## Figures and Tables

**Table 1:** Proteins and peptides identified in brain tumor tissues following the top-down approach.

Uniprot Accession	Protein Name	Amino Acid Position *	[M+H]^+^ Theoretical Monoisotopic	[M+H]^+^ Experimental Monoisotopic	PTMs
P69905	Hemoglobin α-Chain	2-142 (Chain)	15,117.89	15,117.90	-
	Hemoglobin α-Chain des-R _(C-terminal)_	2-141	14,961.79		-
	Hemoglobin α-Chain frag.	2-32	3195.65	3195.66	-
	Hemoglobin α-Chain frag.	2-15	1513.80	1513.80	Oxidation to oxolactone W_15_
P68871	Hemoglobin β-Chain	2-147 (Chain)	15,858.26	15,858.25	-
P63313	Thymosin β10	2-44 (Chain)	4934.53	4934.54	Acetylation _N-terminal_
	Thymosin β10 des-IS [10,11]	2-42	4734.41	4734.42	Acetylation _N-terminal_
	Thymosin β10 des-SEIS	2-40	4518.34	4518.36	Acetylation _N-terminal_
	Thymosin β10 des-RSEIS	2-39	4362.24	4362.24	Acetylation _N-terminal_
	Thymosin β10 frag.	2-27	2964.50	2964.51	Acetylation _N-terminal_
	Thymosin β10 frag.	28-41	1687.89	1687.89	-
P62328	Thymosin β4	2-44 (Chain)	4961.50	4961.50	Acetylation _N-terminal_
	Thymosin β4 sulfoxide	2-44 (Chain)	4977.49	4977.49	Acetylation _N-terminal_ Oxidation M_7_
	Thymosin β4 des-ES _(C-terminal)_	2-42	4745.42	4745.43	Acetylation _N-terminal_
	Thymosin β4 des-AGES _(C-terminal)_	2-40	4617.36	4617.36	Acetylation _N-terminal_
	Thymosin β4 frag.	2-19	2151.10	2151.10	Acetylation _N-terminal_
	Thymosin β4 frag.	2-14	1566.70	1566.70	Acetylation _N-terminal_
	Thymosin β4 frag.	2-12	1304.60	1304.61	Acetylation _N-terminal_
	Thymosin β4 frag.	2-15	1694.79	1694.80	Acetylation _N-terminal_
	Thymosin β4 frag.	2-16	1781.83	1781.83	Acetylation _N-terminal_
P20962	Parathymosin (2-102)	2-102 (Chain)	11,435.17	11,435.15	Acetylation _N-terminal_
	Parathymosin frag. Des-GASA	2-98	11,149.04	11,149.03	Acetylation _N-terminal_
P06454	Prothymosin α1 frag.	2-15	1466.69	1466.69	Acetylation _N-terminal_
	Thymosin α1	2-29	3107.51	3107.52	Acetylation _N-terminal_
	Thymosin α11	2-36	3788.83	3788.54	Acetylation _N-terminal_
P61604	10 kDa Heat Shock Protein	2-102 (Chain)	10,836.85	10,836.87	Acetylation _N-terminal_
P59665	α-Defensin 1 [12,13]	65-94	3440.52	3440.53	Disulfide bonds _(66→94, 68→83, 73→93)_
P59665/6	α-Defensin 2 [12,13]	66-94	3369.48	3369.49	Disulfide bonds _(66→94, 68→83, 73→93)_
P59666	α-Defensin 3 [12,13]	65-94	3484.51	3484.51	Disulfide bonds _(66→94, 68→83, 73→93)_
P56385	ATP Synthase Subunit e, Mitochondrial	2-69 (Chain)	7798.29	7798.30	-
P18859	ATP Synthase Coupling Factor 6, Mitochondrial	33-108 (Chain)	8955.55	8955.48	-
P14854	Cytochrome C Oxidase Subunit 6B1	2-86 (Chain)	10,093.67	10,093.66	Acetylation _N-terminal_ Disulfide bonds _(30→65, 40→54)_
P14136	Glial Fibrillary Acidic Protein	388-432	5206.73	5206.74	-
		388-432	5207.72	5207.74	Deamidation Q_388_ or Citrullination R_390_
		388-432	5208.70	5208.72	Deamidation Q_388_ or Citrullination R_390_ Citrullination R_416_
		398-432	5208.70	5208.72	Citrullination R_406, 416_
		388-432	5209.68	5209.69	Deamidation Q_388_ or Citrullination R_390_ Citrullinations R_406,_ R_416_
		388-431	5075.69	5075.70	-
		388-431	5076.67	5076.69	Citrullination R_390 or 406 or 416_
		388-431	5077.66	5077.67	Deamidation Q_388_ or Citrullination R_390_ Citrullination R_416_
		15-36	2185.13	2185.14	-
		15-38	2385.25	2385.26	-
		15-38	2386.23	2386.24	Citrullination
		15-38	2387.22	2387.22	Citrullinations R_29_, R_36_
		41-59	2052.11	2052,11	-
		398-430	3805.00	3805.01	-
		398-430	3805.99	3805.99	Citrullination R_406_
		398-430	3805.99	3805.99	Deamidation N_407_ or Citrullination R_416_
		398-430	3805.99	3805.99	Citrullination R_416_
		398-430	3806.97	3806.98	Citrullination R_406,_ R_416_
		388-430	4976.62	4976.61	-
		388-430	4977.61	4977.63	Deamidation Q_388_ or Citrullination R_390_
		388-430	4978.59	4978.60	Deamidation Q_388_ or Citrullination R_390_ Citrullination R_416_
		388-430	4979.57	4979.59	Deamidation Q_388_ or Citrullination R_390_ Citrullinations R_406,_ R_416_
		398-432	4035.11	4035.12	-
		398-432	4036.10	4036.11	Citrullinations R_406_ or Deamidation N_407_
		398-432	4036.10	4036.10	Citrullinations R_416_
		398-432	4037.08	4037.08	Citrullinations R_406,_ R_416_
		388-405	2028.07	2028.08	-
		388-405	2029.06	2029.06	Deamidation Q_388_ or Citrullination R_390_
		416-432	2028.02	2028.02	-
		22-36	1463.86	1463.86	-
		388-415	3197.73	3197.74	-
		388-415	3198.72	3198.72	Deamidation Q_388_ or Citrullination R_390_
		406-432	3197.68	3197.68	-
		406-432	3198.66	3198.67	Citrullination R_416_
		406-430	2967.57	2967.57	-
P08670	Vimentin	424-466	4953.53	4953.54	-
		424-466	4954.51	4954.53	Citrullination R_440_ or R_450_ or Deamidation Q_453_
		422-466	5180.66	5180.67	-
		422-466	5181.64	5181.65	Deamidation N_422_ or Citrullination R_424_
		422-466	5182.63	5182.64	Deamidation N_422 or 427_ or Citrullination R_424_ Citrullination R_424 or 450_
		430-466	4225.18	4225.19	-
		430-466 (Rt 23.2 min)	4226.17	4226.18	Citrullination R_440_
		430-466 (Rt 24.0 min)	4226.17	4226.18	Citrullination R_450_
		434-466	3814.93	3814.94	-
		443-466	2777.37	2777.37	-
		443-466	2778.35	2778.37	Deamidation N_456_
		443-466	2778.35	2778.36	Citrullination R_450_ or Deamidation Q_453_
		444-466	2664.29	2664.29	-
		444-466	2665.27	2665.28	Deamidation Q_460_
		444-466	2665.27	2665.27	Citrullination R_450_
		447-466	2322.06	2322.06	-
		447-466	2323.04	2323.05	Citrullination R_450_ or Deamidation Q_453_
		54-69	1565.80	1565.81	-
P07108	AcylCoA Binding Protein	2-87 (Chain)	9950.00	9950.03	Acetylation _N-terminal_
	AcylCoA Binding Protein natural variant M_71_→V	2-87 (Chain)	9918.03	9918.04	Acetylation _N-terminal_
P04271	S100B	2-92 (Chain)	10,618.03	10,618.02	Acetylation _N-terminal_
P06703	S100A6	2-90 (Chain)	10,085.32	10,085.32	Acetylation _N-terminal_
		2-90 (Chain)	10,204.32	10,204.40	Acetylation _N-terminal_ Cysteinylation C_3_
		2-90 (Chain)	10,390.39	10,390.38	Acetylation _N-terminal_ Gluthathionylation C_3_
P01011	α-1-Antichimotrypsin	390-423	4023.18	4023.20	-
		387-423	4352.34	4352.36	-
P01009	α-1-Antitrypsin	384-418	4046.20	4046.21	-
Q16555-2	Dihydropyrimidinase-related protein	521-570	5305.80	5305.81	-
		521-572	5475.91	5475.92	-
P0CG48	Ubiquitin	1-76 (Chain)	8560.63	8560.64	-
	Ubiquitin des-GG _(C-terminal)_	1-74	8446.58	8446.59	-
	Ubiquitin des-RGG _(C-terminal)_	1-73	8290.48	8290.56	-
	Ubiquitin des-LRGG _(C-terminal)_	1-72	8177.40	8177.43	-
P05204	Non-histone chromosomal protein HMG-17	2-90 (Chain)	9258.01	9258.02	Deamidation N_72_
P62805	Histone H4	2-103 (Chain)	11,300.39	11,300.49	Acetylation _N-terminal_ Dimethylation K_21_
		2-103 (Chain)	11,342.40	11,342.36	Acetylation _N-terminal_ Acetylation K_17_ or K_32_ Dimethylation K_21_
Q6FI13	Histone H2A type 2-A	2-130 (Chain)	13,998.87	13,998.91	Acetylation _N-terminal_
Q5QNW6	Histone H2B Type 2-F	2-126 (Chain)	13,781.54	13,781.57	-
P61769	β2-microglobulin	21-119 (Chain)	11,722.78	11,722.78	Disulfide bond _45 ↔ 100_
P14174	Macrophage migration inhibitory factor	2-115 (Chain)	12,338.19	12,338.20	-
P00441	Superoxide dismutase [Cu-Zn]	2-154 (Chain)	15,835.87	15,836.00	Acetylation _N-terminal_, Disulfide bond _58 ↔ 147_
P02511	α-crystallin B	1-175 (Chain)	20,189.44	20,189.44	Acetylation _N-terminal_
P80723	BASP1	2-227 (Chain)	22,760.18	22,759.15	Myristoylation _N-terminal_

* (Chain) indicates the identification of the entire protein chain.

**Table 2:** Analyzed specimen data, including ID, patient age and sex, brain tumor WHO grade, and diagnosis.

Specimen ID	Patient Age (y = Years, m = Months)	Patient Sex	Tumor Grade	Diagnosis
MB1	7 y	M	WHO IV	Medulloblastoma
MB2	5 y 6 m	F	WHO IV	Medulloblastoma
MB3	6 y	M	WHO IV	Medulloblastoma
MB4	16 y	M	WHO IV	Medulloblastoma
MB5	8 y	F	WHO IV	Medulloblastoma
MB6	9 y	F	WHO IV	Medulloblastoma
MB7	20 y	M	WHO IV	Medulloblastoma
MB8	3 y	M	WHO IV	Medulloblastoma
MB9	14 y	M	WHO IV	Medulloblastoma
MB10	10 y	M	WHO IV	Medulloblastoma
MB11	16 y	F	WHO IV	Medulloblastoma
MB12	6 y	F	WHO IV	Medulloblastoma
MB13	0 y 5 m	M	WHO IV	Medulloblastoma
MB14	8 y	F	WHO IV	Medulloblastoma
MB15	8 y	M	WHO IV	Medulloblastoma
MB16	6 y	F	WHO IV	Medulloblastoma
PA1	3 y 6 m	M	WHO I	Pilocytic Astrocytoma
PA2	17 y	M	WHO I	Pilocytic Astrocytoma
PA3	9 y	M	WHO I	Pilocytic Astrocytoma
PA4	4 y	F	WHO I	Pilocytic Astrocytoma
PA5	21y	M	WHO I	Pilocytic Astrocytoma
PA6	12 y	M	WHO I	Pilocytic Astrocytoma
PA7	6 y	M	WHO I	Pilocytic Astrocytoma
PA8	4 y	F	WHO I	Pilocytic Astrocytoma
PA9	0 y 8 m	M	WHO I	Pilocytic Astrocytoma
PA10	11 y	F	WHO I	Pilocytic Astrocytoma
PA11	8 y	M	WHO I	Pilocytic Astrocytoma
PA12	12 y	M	WHO I	Pilocytic Astrocytoma
PA13	8 y	F	WHO I	Pilocytic Astrocytoma
PA14	4 y	M	WHO I	Pilocytic Astrocytoma
PA15	3 y	F	WHO I	Pilocytic Astrocytoma
PA16	18y	F	WHO I	Pilocytic Astrocytoma
EP1	15 y	M	WHO II	Ependymoma
EP2	2 y	M	WHO II	Ependymoma
EP3	8 y	M	WHO II	Ependymoma
EP4	12 y	M	WHO II	Ependymoma
EP5	16 y	F	WHO II	Ependymoma
EP6	8 y	F	WHO III	Ependymoma
EP7	12 y	M	WHO III	Ependymoma
EP8	13 y	M	WHO III	Ependymoma
EP9	0 y 7 m	M	WHO III	Ependymoma
EP10	6 y	M	WHO III	Ependymoma
EP11	1 y	F	WHO III	Ependymoma
EP12	1 y	M	WHO III	Ependymoma
GBM1	7 y	M	WHO IV	Glioblastoma
GBM2	8 y	M	WHO IV	Glioblastoma
GBM3	8 y	M	WHO IV	Glioblastoma
GBM4	11 y	F	WHO IV	Glioblastoma
GBM5	11 y	M	WHO IV	Glioblastoma
GBM6	3 y	F	WHO IV	Glioblastoma

## Data Availability

Data is contained within the article or Supplementary Materials.

## Supplementary Materials

The following supporting information can be downloaded at: https://www.mdpi.com/article/10.3390/ijms23063196/s1.

## References

[1] Petralia F., Tignor N., Reva B., Koptyra M., Chowdhury S., Rykunov D., Krek A., Ma W., Zhu Y., Ji J. (2020). Integrated Proteogenomic Characterization across Major Histological Types of Pediatric Brain Cancer. Cell.

[2] Anagnostopoulos A.K., Tsangaris G.T. (2014). The proteomics of pediatric brain tumors. Expert Rev. Proteom..

[3] Tsangaris G.T., Anagnostopoulos A.K. (2018). Pediatric brain tumors: Update of proteome-based studies. J. Proteom..

[4] Samuel N., Remke M., Rutka J.T., Raught B., Malkin D. (2014). Proteomic analyses of CSF aimed at biomarker development for pediatric brain tumors. J. Neurooncol..

[5] Tsangaris G.T., Anastasoviti M.C., Anagnostopoulos A.K. (2021). Proteomics of pediatric ependymomas: A review. Childs Nerv. Syst..

[6] Desiderio C., Rossetti D.V., Castagnola M., Massimi L., Tamburrini G. (2021). Adamantinomatous craniopharyngioma: Advances in proteomic research. Childs Nerv. Syst..

[7] Iavarone F., Desiderio C., Vitali A., Messana I., Martelli C., Castagnola M., Cabras T. (2018). Cryptides: Latent peptides everywhere. Crit. Rev. Biochem. Mol. Biol..

[8] Martelli C., Iavarone F., D’Angelo L., Arba M., Vincenzoni F., Inserra I., Delfino D., Rossetti D.V., Caretto M., Massimi L. (2015). Integrated proteomic platforms for the comparative characterization of medulloblastoma and pilocytic astrocytoma pediatric brain tumors: A preliminary study. Mol. Biosyst..

[9] Rossetti D.V., Massimi L., Martelli C., Vincenzoni F., Di Silvestre S., Scorpio G., Tamburrini G., Caldarelli M., Urbani A., Desiderio C. (2020). Ependymoma Pediatric Brain Tumor Protein Fingerprinting by Integrated Mass Spectrometry Platforms: A Pilot Investigation. Cancers.

[10] Delfino D., Rossetti D.V., Martelli C., Inserra I., Vincenzoni F., Castagnola M., Urbani A., Scarpa S., Fuso A., Cavallaro R.A. (2019). Exploring the brain tissue proteome of TgCRND8 Alzheimer’s Disease model mice under B vitamin deficient diet induced hyperhomocysteinemia by LC-MS top-down platform. J. Chromatogr. B Analyt. Technol. Biomed. Life Sci..

[11] Martelli C., Serra R., Inserra I., Rossetti D.V., Iavarone F., Vincenzoni F., Castagnola M., Urbani A., Tamburrini G., Caldarelli M. (2019). Investigating the Protein Signature of Adamantinomatous Craniopharyngioma Pediatric Brain Tumor Tissue: Towards the Comprehension of Its Aggressive Behavior. Dis. Markers..

[12] Holla F.K., Postma T.J., Blankenstein M.A., van Mierlo T.J.M., Vos M.J., Sizoo E.M., de Groot M., Uitdehaag B.M.J., Buter J., Klein M. (2016). Prognostic value of the S100B protein in newly diagnosed and recurrent glioma patients: A serial analysis. J. Neurooncol..

[13] Czarnecka A.M., Campanella C., Zummo G., Cappello F. (2006). Heat shock protein 10 and signal transduction: A “capsula eburnea” of carcinogenesis?. Cell Stress Chaperones..

[14] Hannappel E., Huff T. (2003). The thymosins. Prothymosin alpha, parathymosin, and beta-thymosins: Structure and function. Vitam Horm..

[15] Kuzan A. (2016). Thymosin β as an Actin-binding Protein with a Variety of Functions. Adv. Clin. Exp. Med..

[16] Sribenja S., Wongkham S., Wongkham C., Yao Q., Chen C. (2013). Roles and mechanisms of β-thymosins in cell migration and cancer metastasis: An update. Cancer Investig..

[17] Sribenja S., Li M., Wongkham S., Wongkham C., Yao Q., Chen C. (2009). Advances in thymosin beta10 research: Differential expression, molecular mechanisms, and clinical implications in cancer and other conditions. Cancer Investig..

[18] Chen C., Li M., Yang H., Chai H., Fisher W., Yao Q. (2005). Roles of thymosins in cancers and other organ systems. World J. Surg..

[19] Kim Y.Z. (2014). Altered histone modifications in gliomas. Brain Tumor Res. Treat..

[20] Zhang L., Wang D., Han X., Tang F., Gao D. (2019). Mechanism of methylation and acetylation of high GDNF transcription in glioma cells: A review. Heliyon.

[21] Williams M.J., Singleton W.G., Lowis S.P., Malik K., Kurian K.M. (2017). Therapeutic Targeting of Histone Modifications in Adult and Pediatric High-Grade Glioma. Front Oncol..

[22] Fan W., Song Y., Ren Z., Cheng X., Li P., Song H., Jia L. (2020). Glioma cells are resistant to inflammation-induced alterations of mitochondrial dynamics. Int. J. Oncol..

[23] Ostrom Q.T., Rubin J.B., Lathia J.D., Berens M.E., Barnholtz-Sloan J.S. (2018). Females have the survival advantage in glioblastoma. Neuro Oncol..

[24] Yang W., Warrington N.M., Taylor S.J., Whitmire P., Carrasco E., Singleton K.W., Wu N., Lathia J.D., Berens M.E., Kim A.H. (2019). Sex differences in GBM revealed by analysis of patient imaging, transcriptome, and survival data. Sci. Transl. Med..

[25] McCrea H.J., Bander E.D., Venn R.A., Reiner A.S., Iorgulescu J.B., Puchi L.A., Schaefer P.M., Cederquist G., Greenfield J.P. (2015). Sex, Age, Anatomic Location, and Extent of Resection Influence Outcomes in Children With High-grade Glioma. Neurosurgery.

[26] György B., Tóth E., Tarcsa E., Falus A., Buzás E.I. (2006). Citrullination: A posttranslational modification in health and disease. Int. J. Biochem. Cell Biol..

[27] Yuzhalin A.E. (2019). Citrullination in Cancer. Cancer Res..

[28] Jiang Z., Cui Y., Wang L., Zhao Y., Yan S., Chang X. (2013). Investigating citrullinated proteins in tumour cell lines. World J. Surg. Oncol..

[29] Willumsen N., Bager C.L., Leeming D.J., Smith V., Christiansen C., Karsdal M.A., Dornan D., Bay-Jensen A.C. (2014). Serum biomarkers reflecting specific tumor tissue remodeling processes are valuable diagnostic tools for lung cancer. Cancer Med..

[30] Ishigami A., Ohsawa T., Hiratsuka M., Taguchi H., Kobayashi S., Saito Y., Murayama S., Asaga H., Toda T., Kimura N. (2005). Abnormal accumulation of citrullinated proteins catalyzed by peptidylarginine deiminase in hippocampal extracts from patients with Alzheimer’s disease. J. Neurosci. Res..

[31] Jang B., Jin J.K., Jeon Y.C., Cho H.J., Ishigami A., Choi K.C., Carp R.I., Maruyama N., Kim Y.S., Choi E.K. (2010). Involvement of peptidylarginine deiminase-mediated post-translational citrullination in pathogenesis of sporadic Creutzfeldt-Jakob disease. Acta Neuropathol..

[32] Hsu P.C., Liao Y.F., Lin C.L., Lin W.H., Liu G.Y., Hung H.C. (2014). Vimentin is involved in peptidylarginine deiminase 2-induced apoptosis of activated Jurkat cells. Mol. Cells.

[33] Chang X., Han J., Pang L., Zhao Y., Yang Y., Shen Z. (2009). Increased PADI4 expression in blood and tissues of patients with malignant tumors. BMC Cancer.

[34] Chang X., Hou X., Pan J., Fang K., Wang L., Han J. (2011). Investigating the pathogenic role of PADI4 in oesophageal cancer. Int. J. Biol. Sci..

[35] Wang Y., Chen R., Gan Y., Ying S. (2021). The roles of PAD2- and PAD4-mediated protein citrullination catalysis in cancers. Int. J. Cancer.

[36] Jang B., Kim M.J., Lee Y.J., Ishigami A., Kim Y.S., Choi E.K. (2020). Vimentin citrullination probed by a novel monoclonal antibody serves as a specific indicator for reactive astrocytes in neurodegeneration. Neuropathol. Appl. Neurobiol..

[37] Damgaard D., Senolt L., Nielsen M.F., Pruijn G.J., Nielsen C.H. (2014). Demonstration of extracellular peptidylarginine deiminase (PAD) activity in synovial fluid of patients with rheumatoid arthritis using a novel assay for citrullination of fibrinogen. Arthritis Res Ther..

[38] Vossenaar E.R., Radstake T.R., van der Heijden A., van Mansum M.A., Dieteren C., de Rooij D.J., Barrera P., Zendman A.J., van Venrooij W.J. (2004). Expression and activity of citrullinating peptidylarginine deiminase enzymes in monocytes and macrophages. Ann. Rheum. Dis..

[39] Brentville V.A., Vankemmelbeke M., Metheringham R.L., Durrant L.G. (2020). Post-translational modifications such as citrullination are excellent targets for cancer therapy. Semin. Immunol..

[40] Podor T.J., Singh D., Chindemi P., Foulon D.M., McKelvie R., Weitz J.I., Austin R., Boudreau G., Davies R. (2002). Vimentin exposed on activated platelets and platelet microparticles localizes vitronectin and plasminogen activator inhibitor complexes on their surface. J. Biol. Chem..

[41] Moisan E., Girard D. (2006). Cell surface expression of intermediate filament proteins vimentin and lamin B1 in human neutrophil spontaneous apoptosis. J. Leukoc. Biol..

[42] Hill J.A., Southwood S., Sette A., Jevnikar A.M., Bell D.A., Cairns E. (2003). Cutting edge: The conversion of arginine to citrulline allows for a high-affinity peptide interaction with the rheumatoid arthritis-associated HLA-DRB1*0401 MHC class II molecule. J. Immunol..

[43] Ireland J., Herzog J., Unanue E.R. (2006). Cutting edge: Unique T cells that recognize citrullinated peptides are a feature of protein immunization. J. Immunol..

[44] Brentville V.A., Metheringham R.L., Gunn B., Symonds P., Daniels I., Gijon M., Cook K., Xue W., Durrant L.G. (2016). Citrullinated Vimentin Presented on MHC-II in Tumor Cells Is a Target for CD4+ T-Cell-Mediated Antitumor Immunity. Cancer Res..

[45] Brentville V.A., Metheringham R.L., Daniels I., Atabani S., Symonds P., Cook K.W., Vankemmelbeke M., Choudhury R., Vaghela P., Gijon M. (2020). Combination vaccine based on citrullinated vimentin and enolase peptides induces potent CD4-mediated anti-tumor responses. J. Immunother. Cancer.

[46] Benham H., Nel H.J., Law S.C., Mehdi A.M., Street S., Ramnoruth N., Pahau H., Lee B.T., Ng J., Brunck M.E. (2015). Citrullinated peptide dendritic cell immunotherapy in HLA risk genotype-positive rheumatoid arthritis patients. Sci. Transl. Med..

[47] Desiderio C., D’Angelo L., Rossetti D.V., Iavarone F., Giardina B., Castagnola M., Massimi L., Tamburrini G., Di Rocco C. (2012). Cerebrospinal fluid top-down proteomics evidenced the potential biomarker role of LVV- and VV-hemorphin-7 in posterior cranial fossa pediatric brain tumors. Proteomics.

[48] Altinoz M.A., Elmaci I., Ince B., Ozpinar A., Sav A.M. (2016). Hemoglobins, Hemorphins, and 11p15.5 Chromosomal Region in Cancer Biology and İmmunity with Special Emphasis for Brain Tumors. J. Neurol. Surg. A Cent. Eur. Neurosurg..

[49] Marti H.R., Beale D., Lehmann H. (1967). Haemoglobin Koelliker: A new acquired haemoglobin appearing after severe haemolysis: Alpha-2 minus 141 Arg beta-2. Acta Haematol..

[50] Schilirò G., Russo A., Azzia N. (1982). Hemoglobin Koelliker (alpha 2 minus 141 arg beta 2) in favism. Acta Haematol..

[51] Fasan G., Grandgeorge M., Vigneron C., Dellacherie E. (1991). Preparation of unaltered hemoglobin from human placentas for possible use in blood substitutes. J. Biochem. Biophys. Methods.

[52] Michel B., Igić R., Leray V., Deddish P.A., Erdös E.G. (1996). Removal of Arg141 from the alpha chain of human hemoglobin by carboxypeptidases N and M. Circ. Res..

[53] Gittleman H., Ostrom Q.T., Stetson L.C., Waite K., Hodges T.R., Wright C.H., Wright J., Rubin J.B., Berens M.E., Lathia J. (2019). Sex is an important prognostic factor for glioblastoma but not for nonglioblastoma. Neurooncol. Pract..

[54] Jia Y., Buehler P.W., Boykins R.A., Venable R.M., Alayash A.I. (2007). Structural basis of peroxide-mediated changes in human hemoglobin: A novel oxidative pathway. J. Biol. Chem..

[55] Fedorova M., Todorovsky T., Kuleva N., Hoffmann R. (2010). Quantitative evaluation of tryptophan oxidation in actin and troponin I from skeletal muscles using a rat model of acute oxidative stress. Proteomics.

[56] Schöneich C. (2018). Novel chemical degradation pathways of proteins mediated by tryptophan oxidation: Tryptophan side chain fragmentation. J. Pharm. Pharmacol..

[57] Barelli S., Canellini G., Thadikkaran L., Crettaz D., Quadroni M., Rossier J.S., Tissot J.D., Lion N. (2008). Oxidation of proteins: Basic principles and perspectives for blood proteomics. Proteom. Clin. Appl..

[58] Fellers R.T., Greer J.B., Early B.P., Yu X., LeDuc R.D., Kelleher N.L., Thomas P.M. (2015). ProSight Lite: Graphical software to analyze top-down mass spectrometry data. Proteomics.

[59] Szklarczyk D., Gable A.L., Lyon D., Junge A., Wyder S., Huerta-Cepas J., Simonovic M., Doncheva N.T., Morris J.H., Bork P. (2019). STRING v11: Protein–Protein association networks with increased coverage, supporting functional discovery in genome-wide experimental datasets. Nucleic Acids Res..

[60] Mi H., Huang X., Muruganujan A., Tang H., Mills C., Kang D., Thomas P.D. (2017). PANTHER version 11: Expanded annotation data from Gene Ontology and Reactome pathways, and data analysis tool enhancements. Nucleic Acids Res..

