# Supplementary material for: Pediatric Brain Tumors: Signatures from the Intact Proteome
Source: Int J Mol Sci. 2022 Mar 16;23(6):3196. doi: 10.3390/ijms23063196 (PMC8949132; doi:10.3390/ijms23063196)
Supplement: Supplementary file 1 [file ijms-23-03196-s001.zip › ijms-1599857-supplementary.pdf]

A

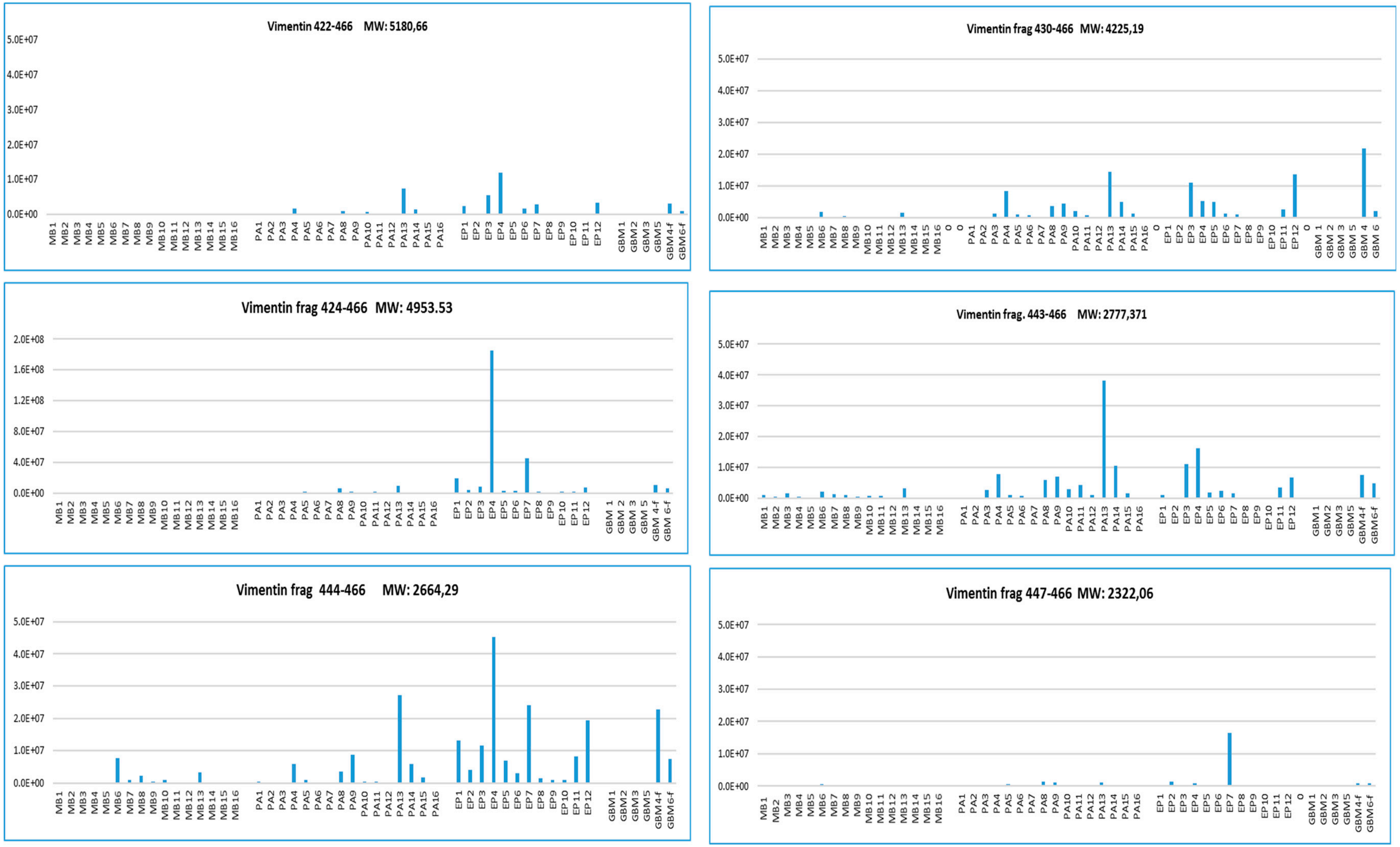

Vimentin 422-466 MW: 5180,66
5.0E+07
4.0E+07
3.0E+07
2.0E+07
1.0E+07
0.0E+00
Vimentin frag 430-466 MW: 4225,19
5.0E+07
4.0E+07
3.0E+07
2.0E+07
1.0E+07
0.0E+00
Vimentin frag 424-466 MW: 4953.53
2.0E+08
1.6E+08
1.2E+08
8.0E+07
4.0E+07
0.0E+00
Vimentin frag. 443-466 MW: 2777,371
5.0E+07
4.0E+07
3.0E+07
2.0E+07
1.0E+07
0.0E+00
Vimentin frag 444-466 MW: 2664,29
5.0E+07
4.0E+07
3.0E+07
2.0E+07
1.0E+07
0.0E+00
Vimentin frag 447-466 MW: 2322,06
5.0E+07
4.0E+07
3.0E+07
2.0E+07
1.0E+07
0.0E+00

B

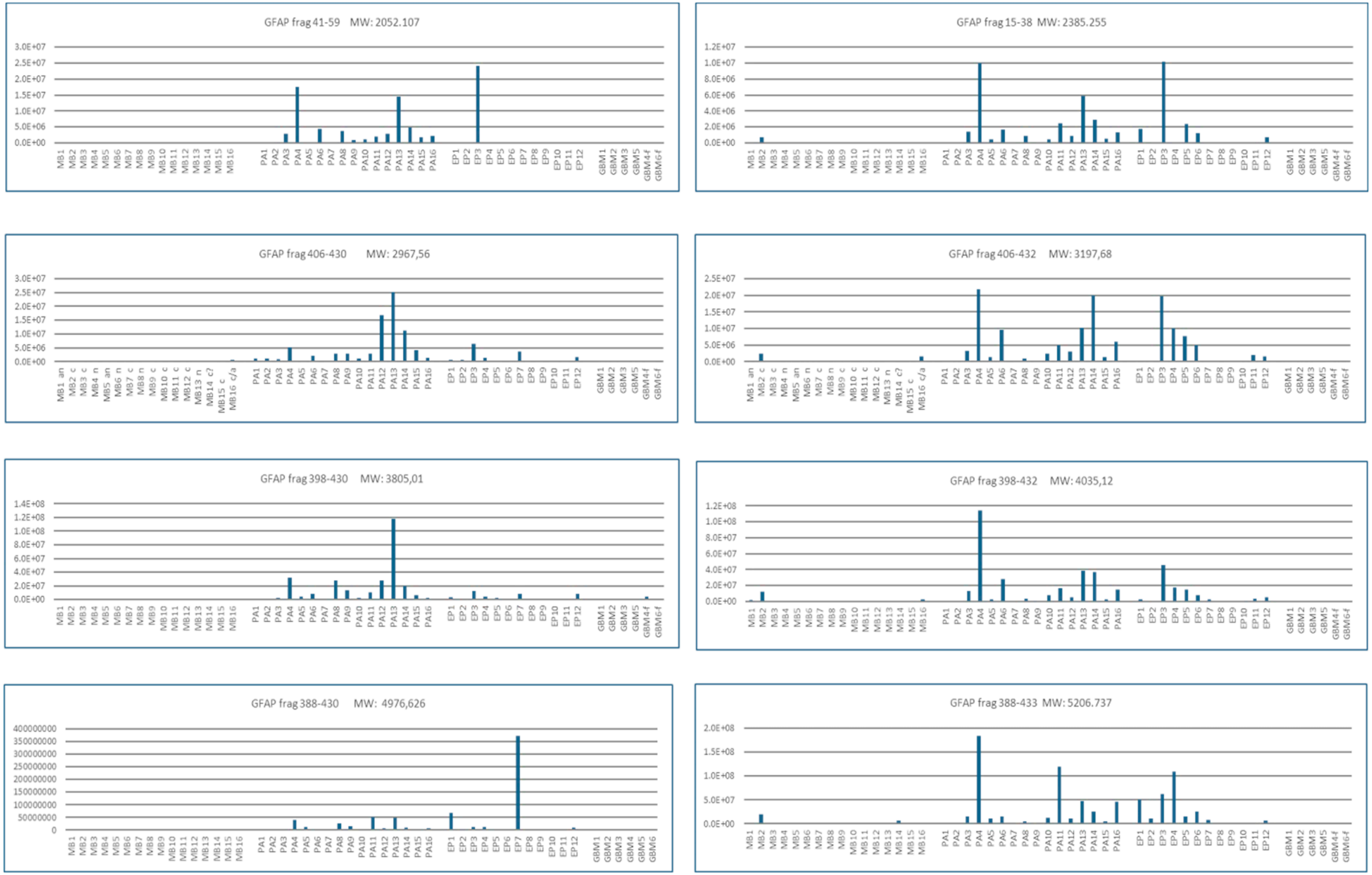


**Supplementary Figure S1.** Distribution of vimentin (panel A) and GFAP (panel B) fragment peptides within the tumor tissue analyzed. In y axis the XIC peak area values are reported.

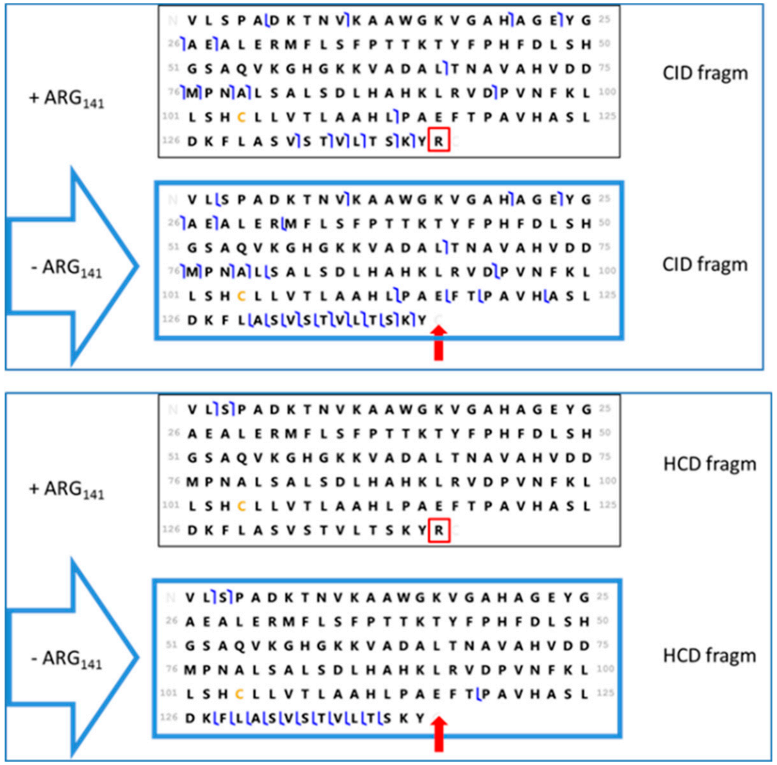


**Supplementary Figure S2.** Sequence coverage data for des-Arg $\alpha$Hb chain identification as resulting by Prosight light comparison of the experimental tandem MS spectra data obtained by CID (upper panels) and HCD (lower panels) fragmentation in comparison to theoretical MS/MS data of both full length and des-Arg $\alpha$Hb chain.
